# The bile acid TUDCA reduces age-related hyperinsulinemia in mice

**DOI:** 10.1038/s41598-022-26915-3

**Published:** 2022-12-23

**Authors:** Lucas Zangerolamo, Marina Carvalho, Leticia Barssotti, Gabriela M. Soares, Carine Marmentini, Antonio C. Boschero, Helena Cristina L. Barbosa

**Affiliations:** Obesity and Comorbidities Research Center, Department of Structural and Functional Biology, University of Campinas, UNICAMP, Campinas, Sao Paulo CEP: 13083-864 Brazil

**Keywords:** Biophysics, Bioenergetics, Ageing, Metabolism, Endocrine system and metabolic diseases

## Abstract

Aging is associated with glucose metabolism disturbances, such as insulin resistance and hyperinsulinemia, which contribute to the increased prevalence of type 2 diabetes (T2D) and its complications in the elderly population. In this sense, some bile acids have emerged as new therapeutic targets to treat TD2, as well as associated metabolic disorders. The taurine conjugated bile acid, tauroursodeoxycholic acid (TUDCA) improves glucose homeostasis in T2D, obesity, and Alzheimer's disease mice model. However, its effects in aged mice have not been explored yet. Here, we evaluated the actions of TUDCA upon glucose-insulin homeostasis in aged C57BL/6 male mice (18-month-old) treated with 300 mg/kg of TUDCA or its vehicle. TUDCA attenuated hyperinsulinemia and improved glucose homeostasis in aged mice, by enhancing liver insulin-degrading enzyme (IDE) expression and insulin clearance. Furthermore, the improvement in glucose-insulin homeostasis in these mice was accompanied by a reduction in adiposity, associated with adipocyte hypertrophy, and lipids accumulation in the liver. TUDCA-treated aged mice also displayed increased energy expenditure and metabolic flexibility, as well as a better cognitive ability. Taken together, our data highlight TUDCA as an interesting target for the attenuation of age-related hyperinsulinemia and its deleterious effects on metabolism.

## Introduction

Aging is considered the largest risk factor for a variety of chronic and metabolic disorders, including type 2 diabetes (T2D), neurodegenerative diseases, cancer, heart disease, and stroke^1^, which significantly impact public health. Furthermore, aging is also linked to the accumulation of fat abdominal and lipids in the liver, both of which are related to insulin resistance and hyperinsulinemia^2^.

Hyperinsulinemia has been frequently associated with aging and is an important etiological factor in the development of metabolic complications^3^. The genesis of hyperinsulinemia in aging remains under debate. It is known that several factors, such as sarcopenia, increased adiposity, and reduced physical activity can contribute to the development of insulin resistance in aging^4^. As a consequence, hyperinsulinemia would appear later as a compensatory mechanism to insulin resistance^5^. Hyperinsulinemia has also been considered as a cause rather than a consequence of insulin resistance, since elevated plasma insulin levels may chronically reduce the number and the activity of the insulin receptors, leading to insulin resistance^5,6^.

Therefore, several strategies have been proposed to reduce age-related hyperinsulinemia and insulin resistance, aiming at to improve the quality of life for the elderly. In this scenario, the tauroursodeoxycholic acid (TUDCA) stands out for presenting broad systemic action. TUDCA is an endogenous bile acid, produced in hepatocytes by the conjugation of the amino acid taurine to ursodeoxycholic bile acid (UDCA), which is present in high concentrations in some species of bears^7^. TUDCA inhibits hepatocytes apoptosis^8^, reduce endoplasmic reticulum (ER) stress in several cell types^9^, and increases β-cell mass in type I diabetes mice model^10^. It is also effective in reducing body weight and ameliorating glucose and insulin tolerance in leptin-deficient Lep^ob/ob^ (ob/ob) mice^9^, diet-induced obese mice^11^, as well as in streptozotocin-induced Alzheimer’s disease (AD) mice model^12,13^.

It has been previously shown that 15 days of TUDCA treatment improves insulin clearance in diet-induced obese mice, by increasing the expression of insulin-degrading enzyme (IDE) in the liver, probably through a mechanism dependent on sphingosine-1-phosphate receptor 2 (S1PR2) activation^11^. In addition, treatment with TUDCA for 30 days reduced plasma insulin concentration in ob/ob mice^9^. These outcomes point to an important role of TUDCA in reducing insulin levels in hyperinsulinemic experimental models. However, the actions of TUDCA on glucose homeostasis in aged mice have not been evaluated yet.

Here, we show that treatment with TUDCA improved glucose-insulin homeostasis in 18-month-old mice, a phenomenon associated with increased insulin clearance and liver IDE expression. These outcomes were accompanied by a reduction in adiposity, adipocyte hypertrophy, and hepatic lipids accumulation. TUDCA-treated Old mice also displayed increased energy expenditure and metabolic flexibility, as well as better cognitive capacity. These findings suggest TUDCA treatment as a promising tool for the control of hyperinsulinemia and its deleterious effects on metabolism in aged individuals.

## Material and methods

### Animal ethic

This study was approved by the Experimental Animal Ethics Committee of the University of Campinas (Protocol No: 5022-1/2018), which was conducted following the last revision of the National Institutes of Health (NIH) guide for the care and use of laboratory animals, and in compliance with the ARRIVE guidelines.

### Establishment of the experimental groups and TUDCA treatment

For this study, male C57BL/6JUnib mice (origin: Zentralinstitut fur Versuchstierzucht—Hannover, Germany) with 3-month-old and 18-month-old, purchased from the animal facility of the University of Campinas, were used. During the experiment procedure, mice were housed in individual cages, on a 12 h light/dark cycle at a controlled temperature of 20–22 °C, with free access to water and a regular rodent diet (Nuvilab CR1, Colombo, PR, Brazil). The 18-month-old mice were randomly divided into two groups, part of which received the bile acid TUDCA (Calbiochem, Sao Paulo, SP, Brazil), intraperitoneally (i.p.), for 20 consecutive days. TUDCA was daily injected (1 injection/day), at a dose of 300 mg/kg body weight^10–13^. The rest of the 18-month-old mice, as well as the 3-month-old mice, received only the TUDCA vehicle, phosphate buffer saline (PBS). Thus, the 3 experimental groups used in this study were formed: (i) 3-month-old mice that were treated with PBS (Ctl group); (ii) 18-month-old mice that were treated with PBS (Old group), and (iii) 18-month-old mice that were treated with TUDCA (Old + TUDCA group).

### Food and water intake and feces production

During the 20 days of treatment with TUDCA or PBS, body weight, food, and water intake were recorded in mice housed individually. A known amount of food and water was placed in each cage, and the determination of the food and water consumption were assessed at 7 a. m. every two days. At the end of the treatments, feces pellets produced by each mouse for 24 h were collected. The fecal pellets were dehydrated at 60 °C until weight stabilized (dry weight).

### Intraperitoneal glucose and insulin tolerance tests (i.p. GTT and i.p. ITT)

At the end of TUDCA or PBS treatment, mice were subjected to i.p. GTT and i.p. ITT. To test glucose tolerance, overnight fasted (12 h) mice were intraperitoneally injected with glucose (2 g/kg body weight). Their blood glucose was measured before (0 min) and 15, 30, 60, 90, and 120 min after glucose load, from the tip of their tails. The area under the curve (AUC) of blood glucose during the GTT was then calculated. To test insulin tolerance, mice were maintained fasted for 4 h before they receive an intraperitoneal administration of 0.75 U/kg insulin (Humulin, Indianapolis, IN, USA), and their blood glucose was measured before (0 min) and 3, 6, 9, 12, 15, and 18 min after insulin administration. The kITT (constant rate for glucose disappearance) was calculated as previously described^14^. In both tests, blood glucose was measured using an Accu-Check glucometer (Roche, Basileia, Switzerland).

### Indirect calorimetry and locomotor activity

At the end of treatments, oxygen consumption (VO_2_), carbon dioxide production (VCO_2_), the respiratory exchange ratio (RER), and energy expenditure (EE) were analyzed using an indirect open-circuit calorimeter (Oxylet system; Pan Lab/Harvard Instruments, Barcelona, Spain). Mice were allowed to adapt for 24 h before data were recorded for the next 24 h (light and dark cycles). For locomotor activity evaluation, mice were individually placed in cages (Multitake Cage LE001; Pan Lab/Harvard Instruments, Barcelona, Spain), and then acclimated for 24 h. The total locomotor activity (in the xy- and z-axes) was registered during 24 h after acclimation using Compulse and Actitrack software (Pan Lab/Harvard Instruments), available at https://www.panlab.com/en/products.

### Determination of interscapular brown adipose tissue (iBAT) and tail temperature

In order to determine the iBAT and tail thermal release in non-anesthetized mice, an infrared camera (FLIR T450sc, FLIR Systems, Inc. Wilsonville, OR, USA) with an infrared resolution of 320 × 240 pixels was used. Images were analyzed using the FLIR Tools 5.1 software (https://www.flir.com.br/products/flir-tools/) according to the manufacturer’s instructions and are presented in the rainbow high-contrast mode that is available in the color palette of the FLIR Tools software.

### Determination of biochemical parameters

At the end of the experimental period, blood glucose was determined in mice after 12 h fasting and 3 h postprandial (fed state), as cited above. Blood samples were also collected from the tip of the tail vein of mice after 12 h fasting for biochemical analyses. The serum samples were obtained by centrifugation of blood samples (1100 g for 15 min at 4 °C) and were stored at − 20 °C for posterior analysis. Total cholesterol (CHOL) and triglycerides (TG) concentrations were measured with colorimetric reagents (Bioclin Quimica Basica Ltda, Belo Horizonte, MG, Brazil; cat No. #K083 and #K117, respectively), following the manufacturer's instructions.

### In vivo insulin clearance

To evaluate the insulin clearance in vivo, at the end of TUDCA or PBS treatments, mice were subjected to 16 h fasting, and blood samples were collected from the tip of the tail before (0 min), 30 and 60 min after a glucose load by gavage (2 g/kg body weight). Blood samples were drawn in heparin capillary tubes and centrifuged at 1100 g for 15 min at 4 °C to obtain plasma. Insulin and c-peptide were measured using specific commercial enzyme-linked immunosorbent assay (ELISA) kits (Chrystal Chem, Inc, Downers Grove, IL, USA; cat No. #90080 and #90050), following the manufacturer's instructions. The c-peptide/insulin ratio was calculated for each time point to determine the insulin clearance, as previously described^15^, and then the AUC was calculated.

### Novel object recognition test (NORT)

In order to evaluate the long-term memory of mice, we performed the novel object recognition test, which is based on the spontaneous tendency of rodents to spend more time exploring novel objects than a familiar one in an open field arena. The test was conducted and analyzed as previously described in detail by us^12^. Briefly, in the habituation phase (first day), mice were habituated to the open field arena in the absence of objects. On the second day (familiarization phase), each mouse was placed in the middle of the arena, containing two identical objects (A and B). On the third day (test phase), one of the familiar objects (B) was replaced for a new object (C) and mice returned to the open field arena and were allowed to explore both objects. In all phases of the test, the animals remain in the arena for 10 min. The total exploration time of the objects during the 10 min, as well as the preference index, were quantified in the familiarization and test phases.

### Terminal experiment and tissue collection

At the end of treatments, mice were anesthetized with isoflurane and naso-anal lengths were measured. Then the mice were sacrificed by decapitation, and the iBAT and liver were collected for posterior analyses. In addition, the epididymal (eWAT), retroperitoneal (rpWAT), inguinal (iWAT), and perirenal (prWAT) adipose tissues, as well as the gastrocnemius skeletal muscle were dissected and weighed. The pancreas was also collected to evaluate insulin secretion, as described below.

### Glucose-stimulated insulin secretion (GSIS) in pancreatic islets

To assess GSIS, the pancreas was digested with collagenase to isolate pancreatic islets, as previously described^16^. Four fresh islets from each mouse were preincubated for 1 h in Krebs–Henseleit buffer solution (KHBS) containing 0.5 g/L bovine serum albumin and 5.6 mM glucose (pH 7.4, with 95% O_2_ and 5% CO_2_ at 37 °C). This medium was then replaced with a fresh buffer, and the islets were incubated for 1 h in the presence of 2.8- or 11.1-mM glucose. After this incubation, the supernatants were collected to access insulin secretion and the remaining islets were homogenized in an alcohol-acid solution to measure total insulin content, using the Ultra-Sensitive Mouse Insulin ELISA Kit (Crystal Chem, Elk Grove Village, IL, USA; cat No. #90080).

### IDE activity assay

IDE activity was measured in liver samples by SensoLyte 520 IDE Activity Assay Kit (AnaSpec, Fremont, Canada; cat No. AS‐72231) following the manufacturer’s instructions. Total IDE activity was calculated as previously described^15^ and was normalized per μg of total protein content determined using the Bradford reagent.

### Liver lipids extraction and quantification

Liver lipids were extracted from 100 mg of tissue homogenized with a 20-fold excess of a chloroform–methanol (2:1 by volume) mixture, as described by Folch^17^. The homogenates were filtered using filter paper, then lipid extracts were dried, and the pellet was dissolved in isopropanol solution. Liver concentrations of CHOL and TG were determined using commercial kits (Bioclin Quimica Basica Ltda, Belo Horizonte, MG, Brazil; cat No. #K083 and #K117, respectively), and were normalized per milligram of liver weight.

### Histological analysis of adipose tissue

For histological analysis, eWAT samples were dissected and immediately fixed in formalin 10%. After 48 h, the samples were dehydrated with a gradient ethanol series and embedded with paraffin for preparing 5 μm sections. The slides were dewaxed, hydrated, and stained with hematoxylin and eosin (H&E). The sections were observed in a light microscope (Olympus BX51, Olympus Corporation, Tokyo, Japan) and the images were captured using the Olympus DP71 (Olympus Corporation, Tokyo, Japan) digital microscope camera at 20 × magnification. To measure adipocyte number and area, 8 random fields per mouse were evaluated using the plugin Adiposoft 1.16 (https://imagej.net/Adiposoft) from ImageJ software (Bethesda, MD, USA), available at https://imagej.nih.gov/ij/download.html.

### RNA extraction and quantitative real-time PCR analysis

The RNA extraction in iBAT and liver samples was performed using 1 mL Trizol® Reagent (Invitrogen, Thermo Fisher Scientific Inc, Waltham, MA, USA; cat. No. 15596026), following phenol–chloroform RNA extraction, according to the manufacturer’s instructions. RNA concentration was measured by Nanodrop (Nanodrop Thermo Scientific, Wilmington, DE, USA), and the purity and integrity of RNAs were determined by the 260/280 nm absorbance ratio. Two μg of total RNA was used for cDNA synthesis, using the High-Capacity cDNA Reverse Transcription Kit (Applied Biosystems, Thermo Fisher Scientific Inc, Waltham, MA, USA). Expression of genes of interest was analyzed by qPCR on 7500 Fast Real-time PCR System (Applied Biosystems), using Fast SYBR Green Master Mix (Applied Biosystems). In all cases, reactions were performed in a final volume of 10 µL. The specificities of amplifications were verified by melting-curve analyses. The relative expression of RNAs was determined after normalization with glyceraldehyde-3-phosphate dehydrogenase (GAPDH), using the 2^−ΔΔCT^ method. Primer pairs used are shown in Supplementary Table S1 and were designed and purchased from IDT- Integrated DNA Technologies.

### Western blot analysis

Western blot analysis was performed as previously detailed^18^. Briefly, liver samples were homogenized in lysis buffer, and protein concentrations were determined using the Bradford method. Thirty-microgram samples of protein lysate were resolved on 10% SDS–polyacrylamide gels and transferred to nitrocellulose membranes, in a Trans-Blot transfer with tris/glycine buffer. The membranes were blocked with 5% albumin for 1 h at room temperature and incubated overnight at 4 °C with primary antibody against IDE (1:1000, Abcam cat. ab32216). After incubation, the appropriate horseradish peroxidase-conjugated secondary antibody (dilution 1:10,000; Thermo Fisher Scientific cat. #31460) was added and the membranes were incubated for 1 h at room temperature. An antibody against GAPDH was used as an internal control (1:10,000, Sigma Aldrich, cat. G9545). The intensities of the protein bands were detected using the Amersham Imager 600 (GE Healthcare Life Sciences, Buckinghamshire, UK), and quantification was performed using densitometry (ImageJ, version 1.8.0_172, Bethesda, MD, USA), available at https://imagej.nih.gov/ij/download.html.

### Statistical analysis

Results are displayed as the mean ± standard error of the mean (SEM) for the number (n) of mice/samples indicated in the figure’s legends. The area under the curve was calculated by trapezoidal integration. The data were analyzed by one-way analysis of variance (ANOVA) followed by the Tukey post-hoc-test, using GraphPad Prism version 6.00 software (GraphPad Inc., CA, USA) (http://www.graphpad.com), and were considered significantly different if the p-value was equal or lower than 0.05 (p ≤ 0.05).

## Results

### TUDCA treatment reduces body weight in old mice

**Figure 1:**
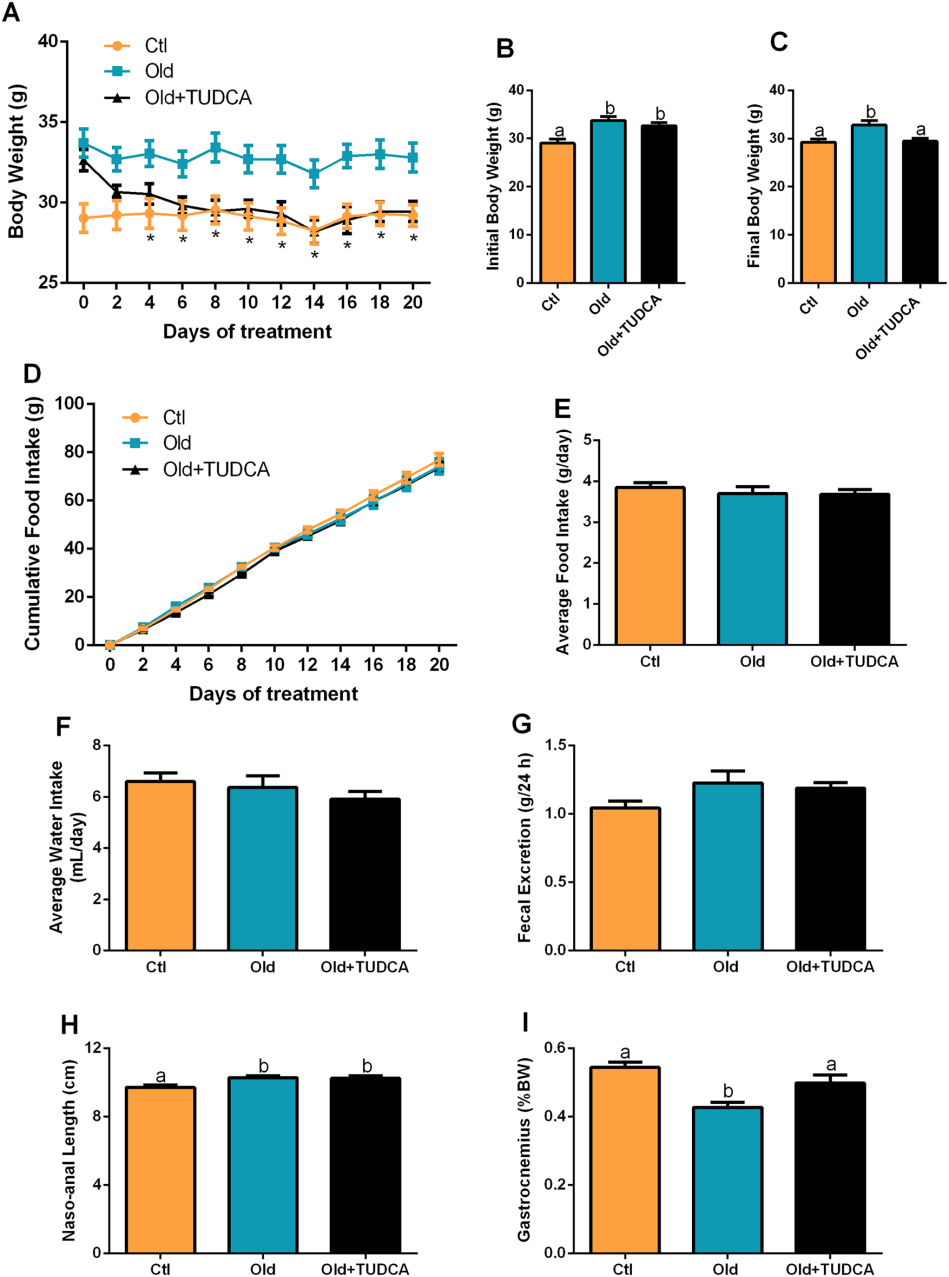
Effects of TUDCA treatment upon body and food parameters. Body weight over the treatment (**A**), initial (**B**), and final (**C**) body weight. Cumulative food intake over 20 days (**D**), and average daily food (**E**) and water (**F**) intake. Fecal excretion during 24 h (**G**). Naso-anal length (**H**), and gastrocnemius weight (**I**). Data are expressed as means ± SEM (n = 6–10). Statistical analysis was performed using the one-way ANOVA test, followed by Tukey post-hoc-test. Letters shared in common between groups indicate no significant difference. Different letters (a and b) indicate statistical difference between groups and * indicate Old is different from Ctl and OLD + TUDCA mice (P ≤ 0.05).

As expected, Old mice displayed higher body weight during, and at the end of the experimental period (Fig. 1A–C). TUDCA significantly reduced body weight from the fourth day of treatment (Fig. 1A), that was maintained until the time of euthanasia (Fig. 1C). In order to evaluate whether the reduced body weight observed in the Old + TUDCA group was due to decreased food and water consumption, these parameters were measured throughout the experimental period, however, no statistical differences were observed between groups (Fig. 1D–F). Analysis of the fecal excretion of these mice indicated no differences between the groups (Fig. 1G), suggesting that changes in fecal production should not be involved with the reduction in body weight observed in Old + TUDCA mice. Even though the Old and Old + TUDCA mice present similar naso-anal lengths (Fig. 1H), the Old + TUDCA mice had a higher gastrocnemius muscle weight compared to the PBS-treated Old mice (Fig. 1I).

### TUDCA treatment decreases adiposity and liver lipids accumulation in old mice

**Figure 2:**
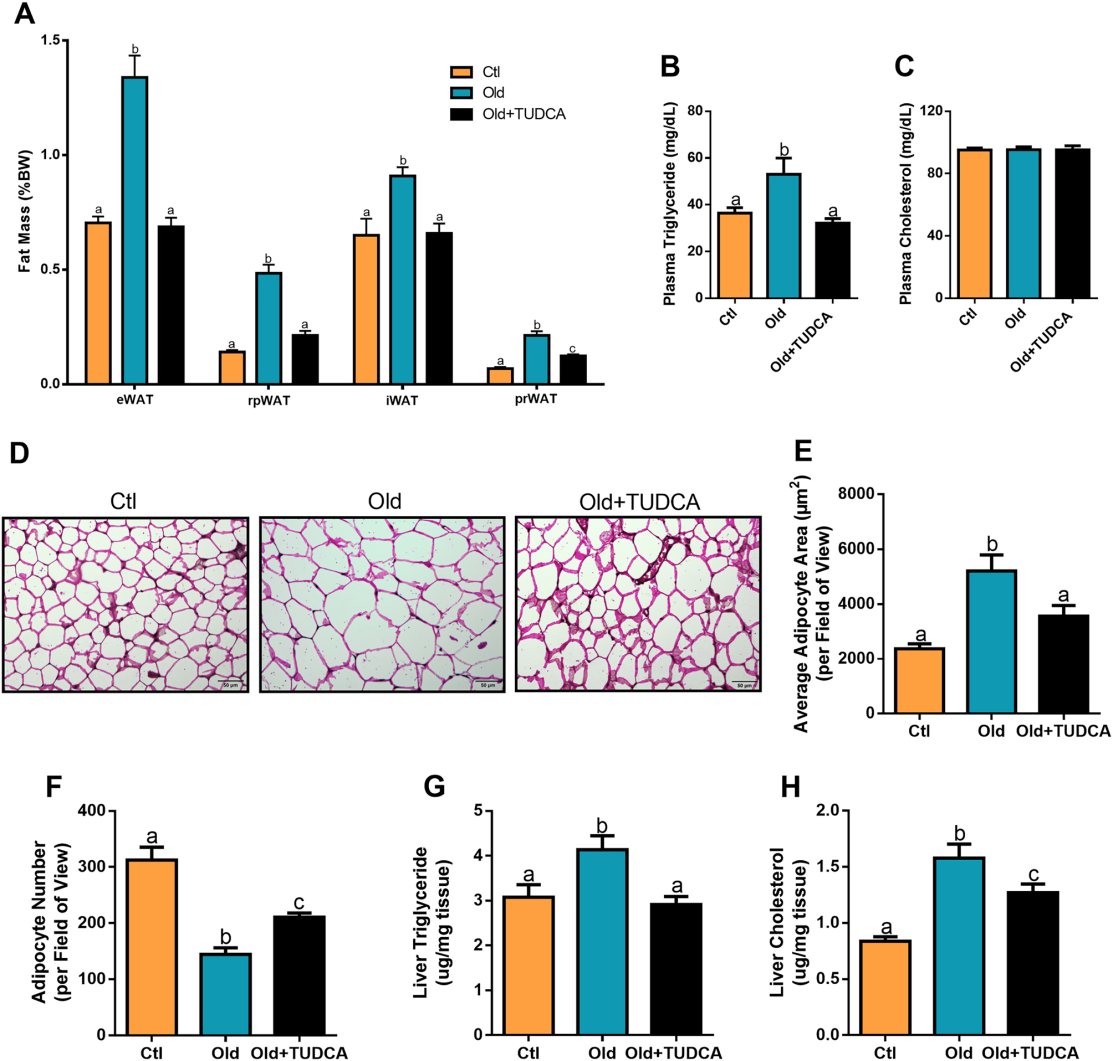
Effects of TUDCA treatment upon fat depots and liver lipids accumulation. Epididymal (eWAT), retroperitoneal (rpWAT), inguinal (iWAT), and perirenal (prWAT) fat pads weight (**A**). Serum triglyceride (**B**) and cholesterol (**C**). H&E sections of Ctl, Old, and Old + TUDCA in eWAT (**D**) (scale bar 50 µm, 20 × magnification). Epididymal WAT adipocyte area (**E**) and number (**F**). Liver triglyceride (**G**) and cholesterol (**H**) content. Data are presented as mean ± SEM (n = 5–10). Statistical analysis was performed using the one-way ANOVA test, followed by Tukey post-hoc-test. Letters shared in common between groups indicate no significant difference. Different letters (a, b, and c) indicate statistical difference between groups (P ≤ 0.05).

Next, we evaluated whether TUDCA treatment caused any repercussion on the mice's fat depots. Old mice showed increased eWAT, rpWAT, iWAT, and prWAT fat pads weight (Fig. 2A), accompanied by an increase in free serum TG (Fig. 2B), with no changes in serum CHOL levels (Fig. 2C), compared with the Ctl group. Meanwhile, following the reductions in body weight, TUDCA treatment also reduced fat accumulation, as well as serum TG levels (Fig. 2A,B). These results were supported by histological and quantitative analyses of the eWAT tissue. We observed hypertrophic adipocytes in the eWAT of Old mice, which resulted in a lower number of adipocytes per field when compared to the Ctl group. In contrast, Old + TUDCA mice displayed smaller area of these adipocytes, similar to that observed in the Ctl group (Fig. 2D–F). In addition, hepatic TG and CHOL levels were increased in Old mice (Fig. 2G,H), indicating excessive liver lipids accumulation. However, these conditions were counteracted by TUDCA treatment (Fig. 2G,H).

### TUDCA treatment improves glucose metabolism in Old mice

**Figure 3:**
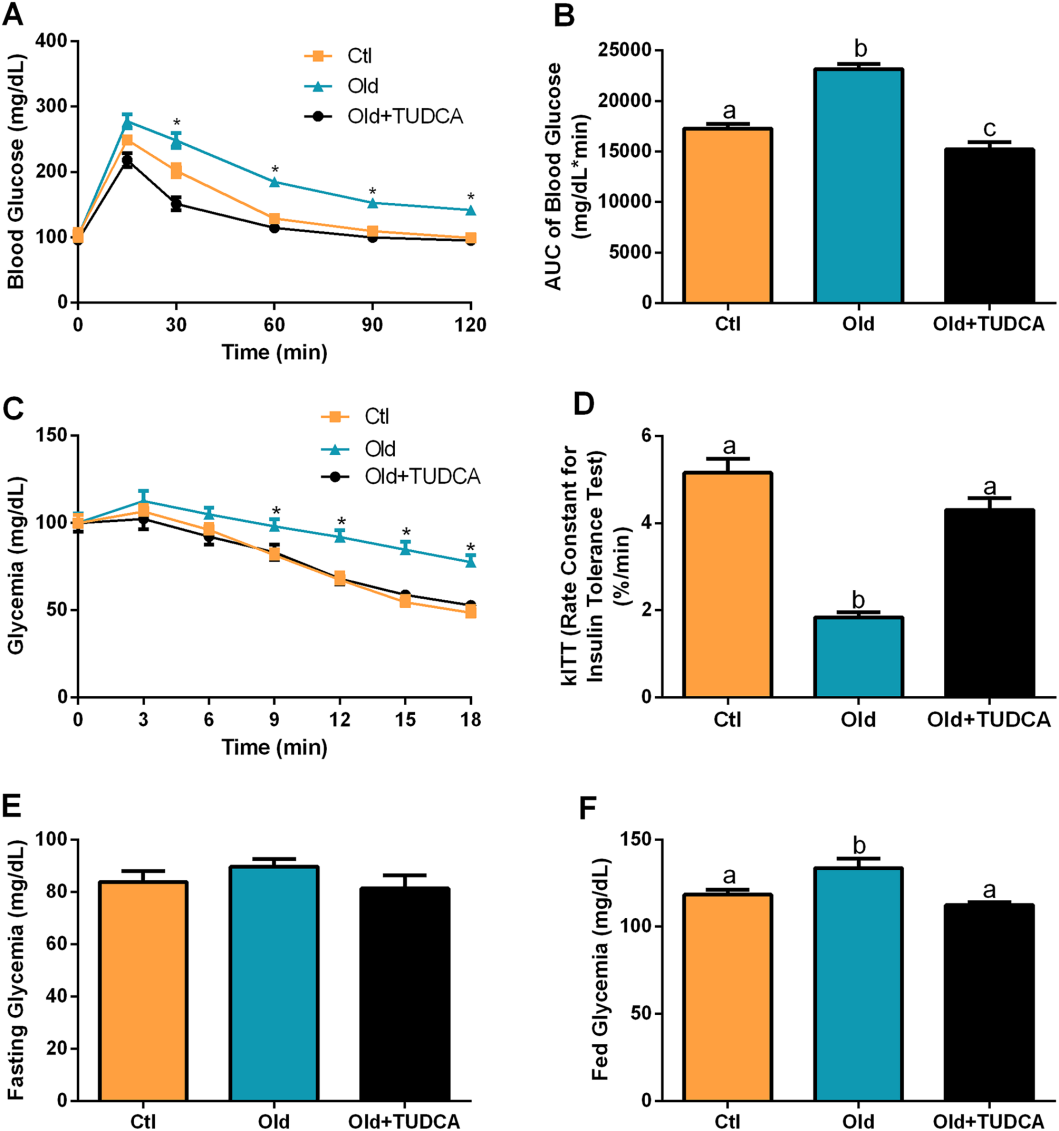
Effects of TUDCA treatment upon glucose tolerance and insulin sensitivity. Blood glucose (**A**) and AUC of blood glucose (**B**) during the i.p. GTT. Blood glucose (**C**) and rate constant for glucose disappearance (kITT) (**D**) during the i.p. ITT. Fasting (**E**) and fed (**F**) glycemia. Data are presented as mean ± SEM (n = 8–10). Statistical analysis was performed using the one-way ANOVA test, followed by Tukey post-hoc-test. Letters shared in common between groups indicate no significant difference. Different letters (a, b, and c) indicate statistical difference between groups and * indicate Old is different from Ctl and OLD + TUDCA mice (P ≤ 0.05).

As expected, Old mice presented an impairment in glucose tolerance (Fig. 3A), as judged by the higher AUC of blood glucose during GTT, compared with Ctl mice (Fig. 3B). Besides that, reduced insulin tolerance, as judged by the lower kITT, was also observed in the Old group (Fig. 3C,D). The Old mice also displayed higher blood glucose levels in the fed state, than those observed in Ctl mice, with no significant differences in fasting glycemia (Fig. 2E,F). After 20-days of TUDCA treatment, an improvement in glucose tolerance (Fig. 3A,B), insulin sensitivity (Fig. 3C,D), and fed glycemia (Fig. 2F) were observed in the Old mice.

### TUDCA treatment increases insulin clearance and does not alter glucose-stimulated insulin secretion in Old mice

**Figure 4:**
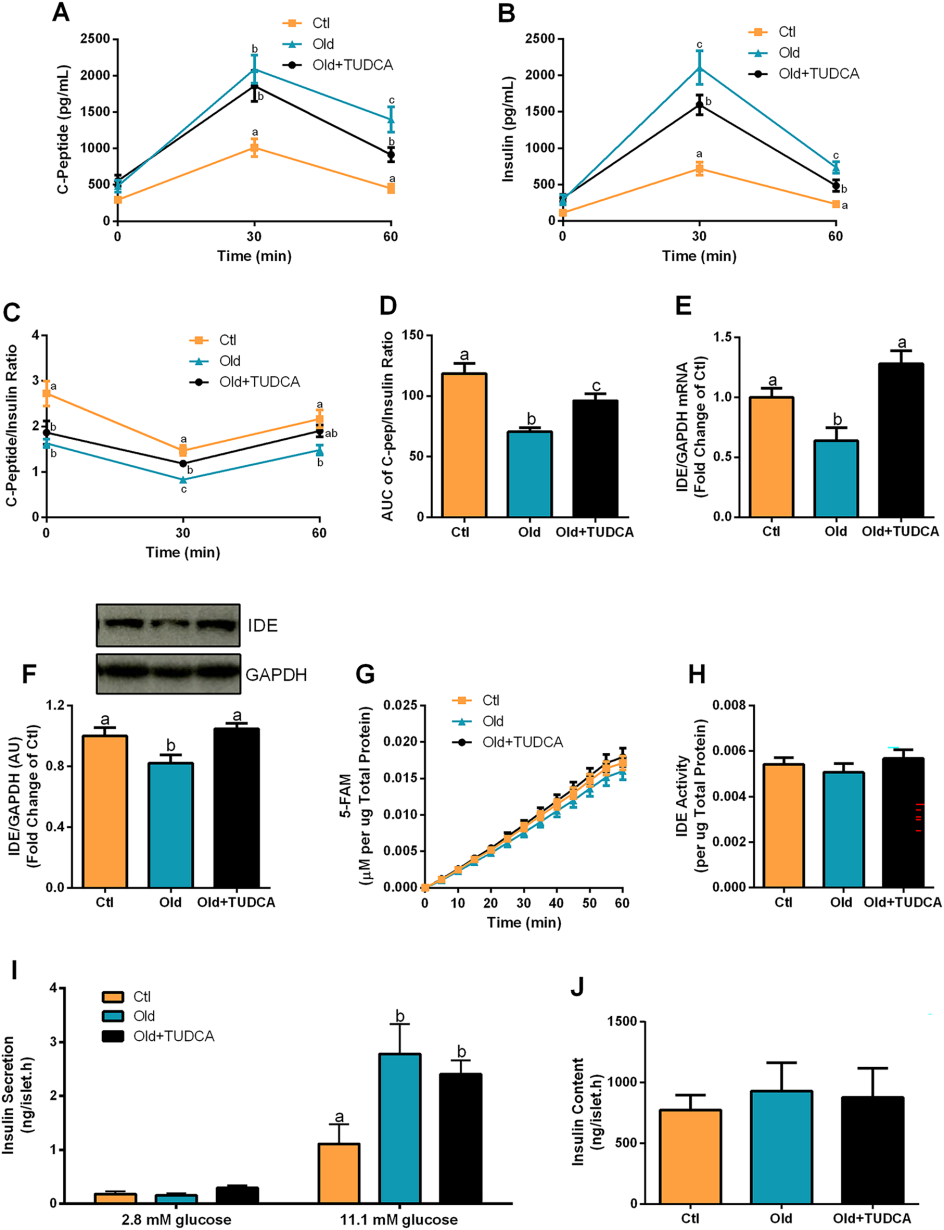
Effects of TUDCA treatment upon insulin clearance and secretion. Plasma c-peptide (**A**), insulin (**B**), and c-peptide/insulin ratio (**C**) before (0 min), 30 and 60 min after the glucose administration, and AUC of plasma c-peptide/insulin ratio (**D**). Gene expression of IDE (**E**), and protein content of IDE (**F**) in the liver, both normalized by GAPDH. Kinetic of the IDE activity assay (**G**) and total IDE activity (**H**) in the liver. Fluorescent intensity at Ex/Em = 490/520 nm was continuously recorded, every 5 min, for 60 min. 5-FAM concentration was calculated using a standard curve and normalized per μg of total protein in the liver. Insulin secretion of isolated pancreatic islets after 1 h incubation with 2.8 and 11.1 mM glucose (**I**) and total insulin content per islet (**J**). Data are presented as mean ± SEM (n = 5–10). Statistical analysis was performed using the one-way ANOVA test, followed by Tukey post-hoc-test. Letters shared in common between groups indicate no significant difference. Different letters (a, b, and c) indicate statistical difference between groups (P ≤ 0.05). *AU* arbitrary units.

Increased plasmatic c-peptide and insulin levels were observed in Old and Old + TUDCA mice, 30 and 60 min after a glucose load (Fig. 4A,B), compared with the Ctl group. However, Old mice presented even higher plasma insulin concentrations 30 min after glucose injection than Old + TUDCA mice, although they had similar c-peptide concentrations at that time (Fig. 4A,B). Pancreatic β-cells co-secrete insulin and c-peptide in a 1:1 ratio, however, the half-life of c-peptide is longer than that of insulin, so, changes in this ratio indicate an alteration in the insulin clearance. A reduction in insulin clearance was observed in Old mice (Fig. 4B,C), as judged by their lower AUC of plasma c-peptide/insulin ratio, compared with the Ctl’s ratio (Fig. 4D). Otherwise, TUDCA-treated Old mice displayed an increase in insulin clearance, compared to the Old mice treated with the vehicle (Fig. 4C,D). These results suggest that insulin secretion was not altered by TUDCA, since plasmatic c-peptide was similar between Old and Old + TUDCA at 30 min, and points TUDCA as a booster of insulin clearance. Then, a question was raised whether TUDCA treatment increases liver expression of IDE, the main enzyme responsible for insulin degradation^19^. Interestingly, the Old + TUDCA mice showed an increase in gene expression (Fig. 4E), as well as in the protein content (Fig. 4F) of IDE in the liver, compared to the non-treated Old mice. Original unprocessed blot images are shown in Supplementary Fig. S1. However, no significant differences in the IDE activity were observed between groups (Fig. 4G,H). Corroborating the similar plasma c-peptide levels observed 30 min after the glucose load, GSIS was also not significantly different in isolated pancreatic islets from Old and Old + TUDCA mice, when incubated at the stimulatory concentration of 11.1 mM glucose (Fig. 4I). In addition, islets insulin content was similar between the groups (Fig. 4J). Taken together, these data indicate that the bile acid TUDCA attenuates hyperinsulinemia in Old mice by increasing liver IDE expression, and consequently, insulin clearance.

### TUDCA treatment increases RER and EE in old mice

**Figure 5:**
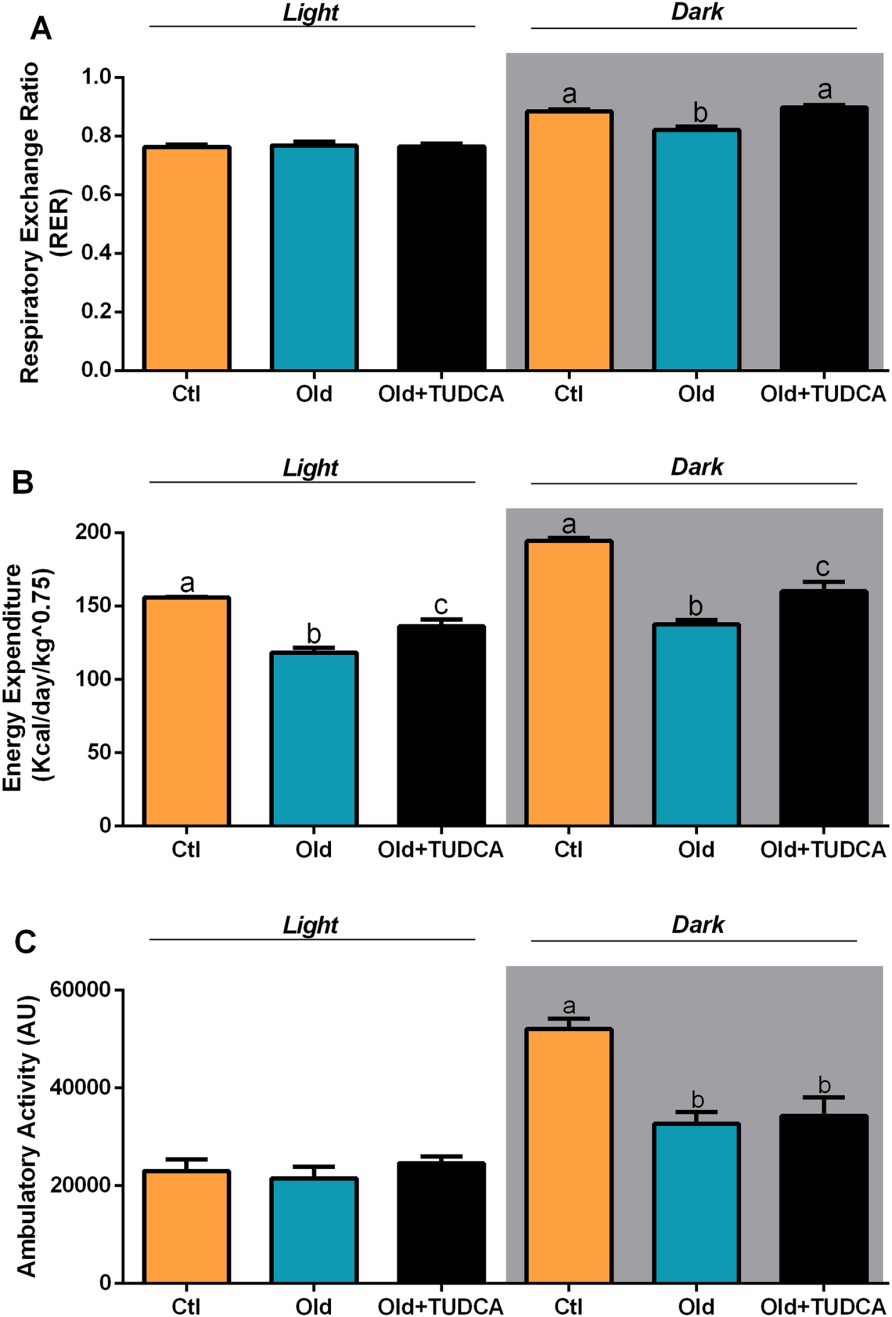
Effects of TUDCA treatment upon RER, EE, and ambulatory activity. Respiratory exchange ratio (RER) (**A**), energy expenditure (EE) (**B**), and ambulatory activity (**C**) average during light (7 a.m. to 6:59 p.m.) and dark (7 p.m. to 6:59 a.m.) cycles. Data are presented as mean ± SEM (n = 6–8). Statistical analysis was performed using the one-way ANOVA test, followed by Tukey post-hoc-test. Letters shared in common between groups indicate no significant difference. Different letters (a, b, and c) indicate statistical difference between groups (P ≤ 0.05).

Next, we evaluated whether the improvement in glucose metabolism observed in Old mice treated with TUDCA modulated RER and EE values in these mice. For this purpose, the animals were submitted to indirect calorimetry. The RER was calculated by measuring the amount of carbon dioxide (CO_2_) produced in comparison to the amount of oxygen (O_2_) used, allowing to predict which substrate is being oxidized and used as a fuel^20^. During the light cycle, when mice are less active, the RER is ∼0.7, indicating a predominant use of fatty acid. In contrast, during the dark cycle, when they are more active and fed, the RER is ∼1.0, suggesting predominantly carbohydrate oxidation^20^. We observed lower RER values in Old mice during the dark cycle, evidencing a lower carbohydrate oxidation capacity, as opposed to the Ctl mice that displayed metabolic flexibility, as judged by their capacity to efficiently increase the values of RER during the dark cycle (Fig. 5A). In addition, Old mice also presented reduced values of EE in both light and dark cycles (Fig. 5B) than those observed in Ctl mice. In contrast, Old mice treated with TUDCA showed an improvement in the values of RER and EE (Fig. 5A,B), suggesting ameliorated metabolic flexibility and energy dissipation. Therefore, increased insulin sensitivity observed in the Old + TUDCA group may increase glucose uptake, contributing to reduced glycemia, and carbohydrate oxidation. In order to assess whether the elevated EE values observed in Old + TUDCA mice were associated with increased locomotion, spontaneous locomotor activity was also assessed in the mice. However, no differences were observed in locomotor activity between the Old and Old + TUDCA mice (Fig. 5C), suggesting that the higher EE observed in the Old + TUDCA group is not associated with increased locomotion.

### TUDCA treatment reverses age-related memory loss in old mice

**Figure 6:**
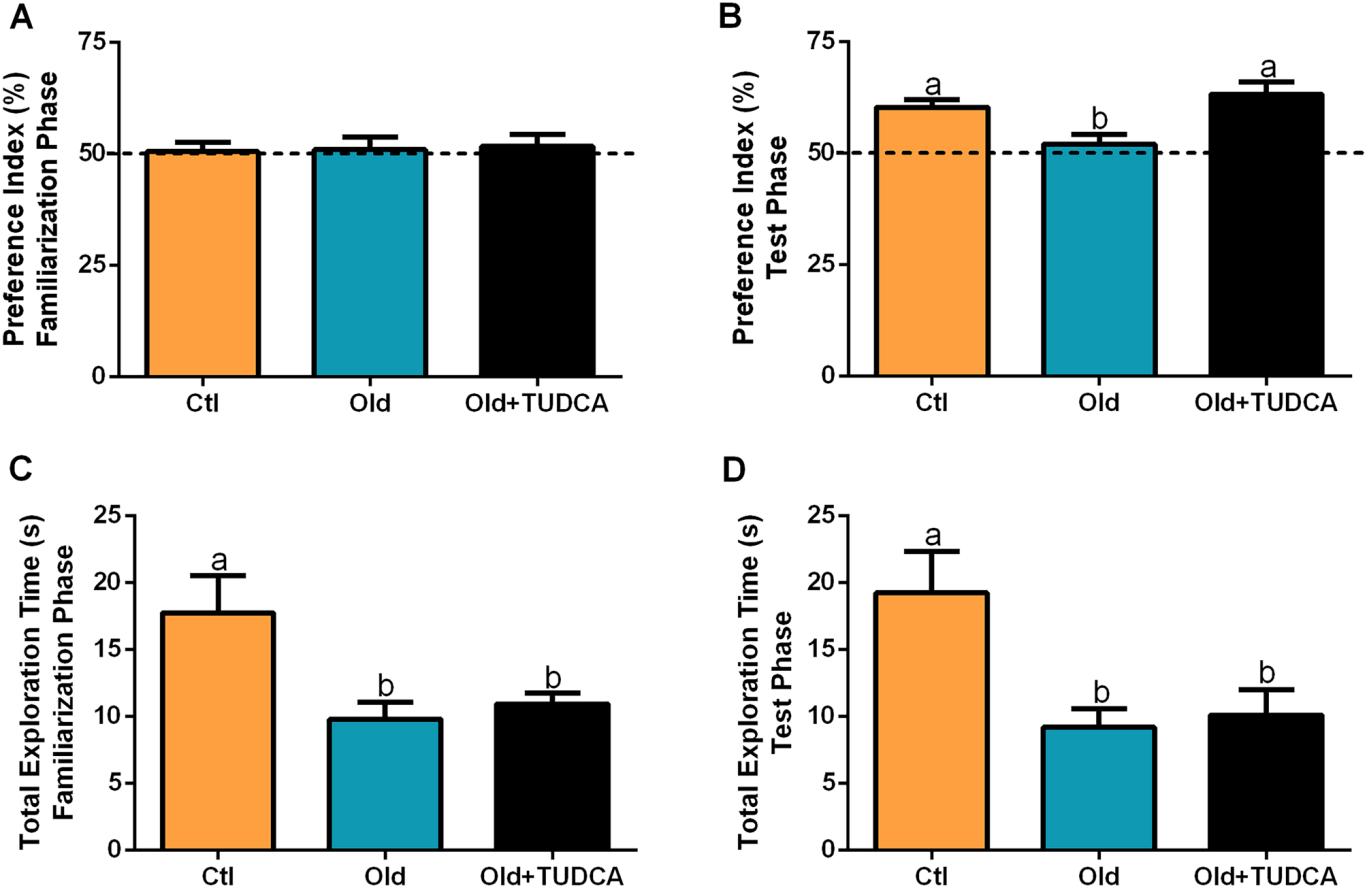
Effects of TUDCA treatment on long-term memory. Preference index (%) during the familiarization (**A**) and test (**B**) phases in the novel object recognition test (NORT). Total exploration time (seconds) during the familiarization (**C**) and test (**D**) phases in the NORT. Data are presented as mean ± SEM (n = 7–10). Statistical analysis was performed using the one-way ANOVA test, followed by Tukey post-hoc-test. Letters shared in common between groups indicate no significant difference. Different letters (a and b) indicate statistical difference between groups (P ≤ 0.05).

Since there is evidence that alterations in glucose metabolism are involved with cognitive decline^12,21^, we subjected the mice to the novel object recognition behavioral test, to assess long-term memory. As expected, all groups displayed a similar preference index for objects during the familiarization phase (Fig. 6A). However, in the test phase, Old mice showed a lower preference index for the new object (Fig. 6B), suggesting impaired memory formation. The treatment with TUDCA improved cognitive ability in Old mice, as judged by better performance during the test phase (Fig. 6B). Besides that, no differences were found between Old and Old + TUDCA groups in the total time of object exploration during the different phases (Fig. 6C,D), suggesting that there was no aversion to objects or deficits in locomotion among these mice.

### TUDCA treatment does not modulate the expression of thermogenesis markers in BAT of old mice

**Figure 7:**
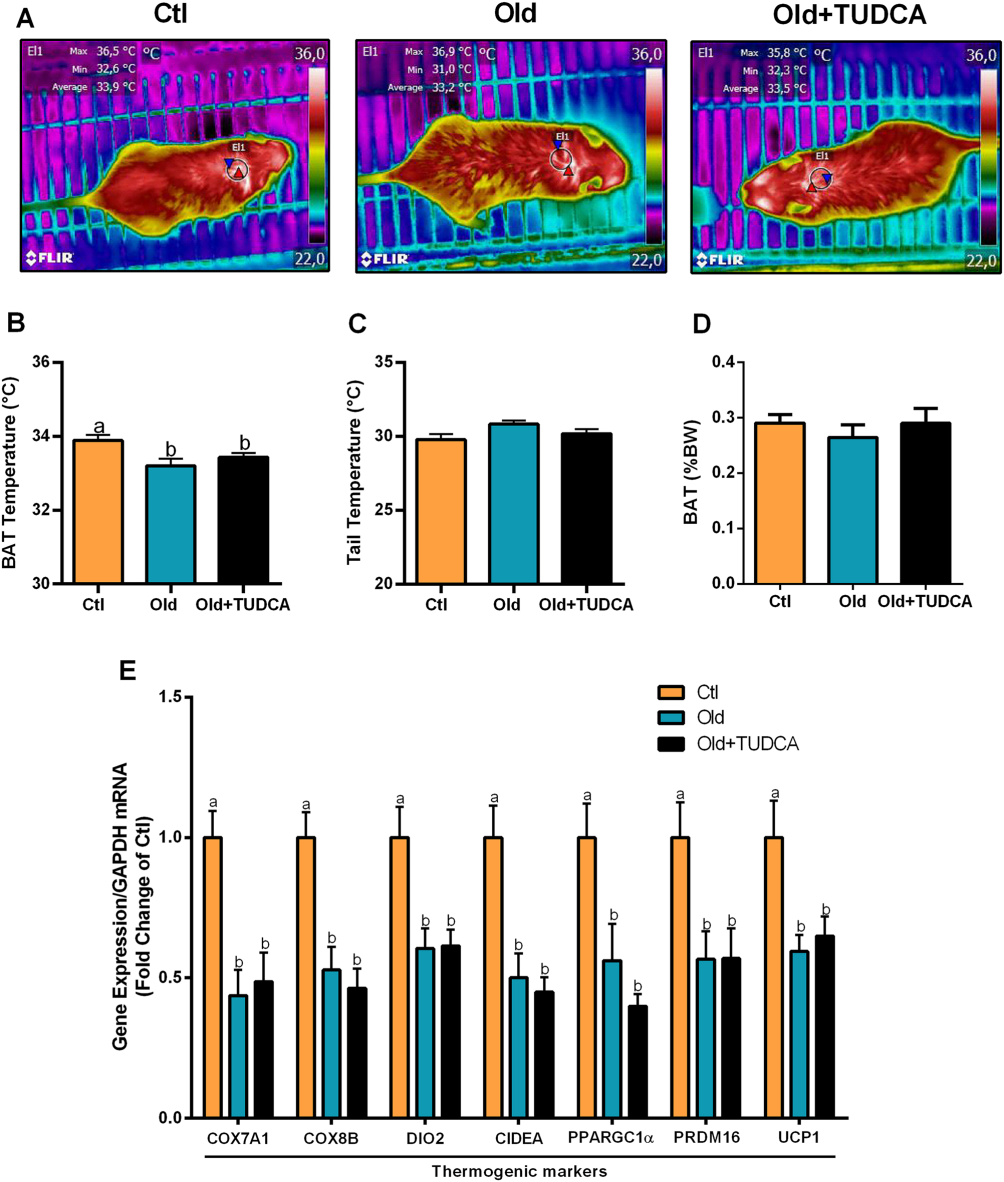
Effects of TUDCA treatment upon temperature and expression of thermogenesis markers in BAT. Snapshots of the dorsal region were obtained using a thermographic camera for the determination of the temperature in the region corresponding to BAT (**A**,**B**) and tail (**C**). Interscapular BAT fat pad weight (**D**). Real-time PCR analysis of COX7A1, COX8B, DIO2, CIDEA, PPARGC1α, PRDM16, and UCP1 (**E**), normalized by GAPDH, in BAT. Data are presented as mean ± SEM (n = 6–10). Statistical analysis was performed using the one-way ANOVA test, followed by Tukey post-hoc-test. Letters shared in common between groups indicate no significant difference. Different letters (a and b) indicate statistical difference between groups (P ≤ 0.05).

**Figure 8:**
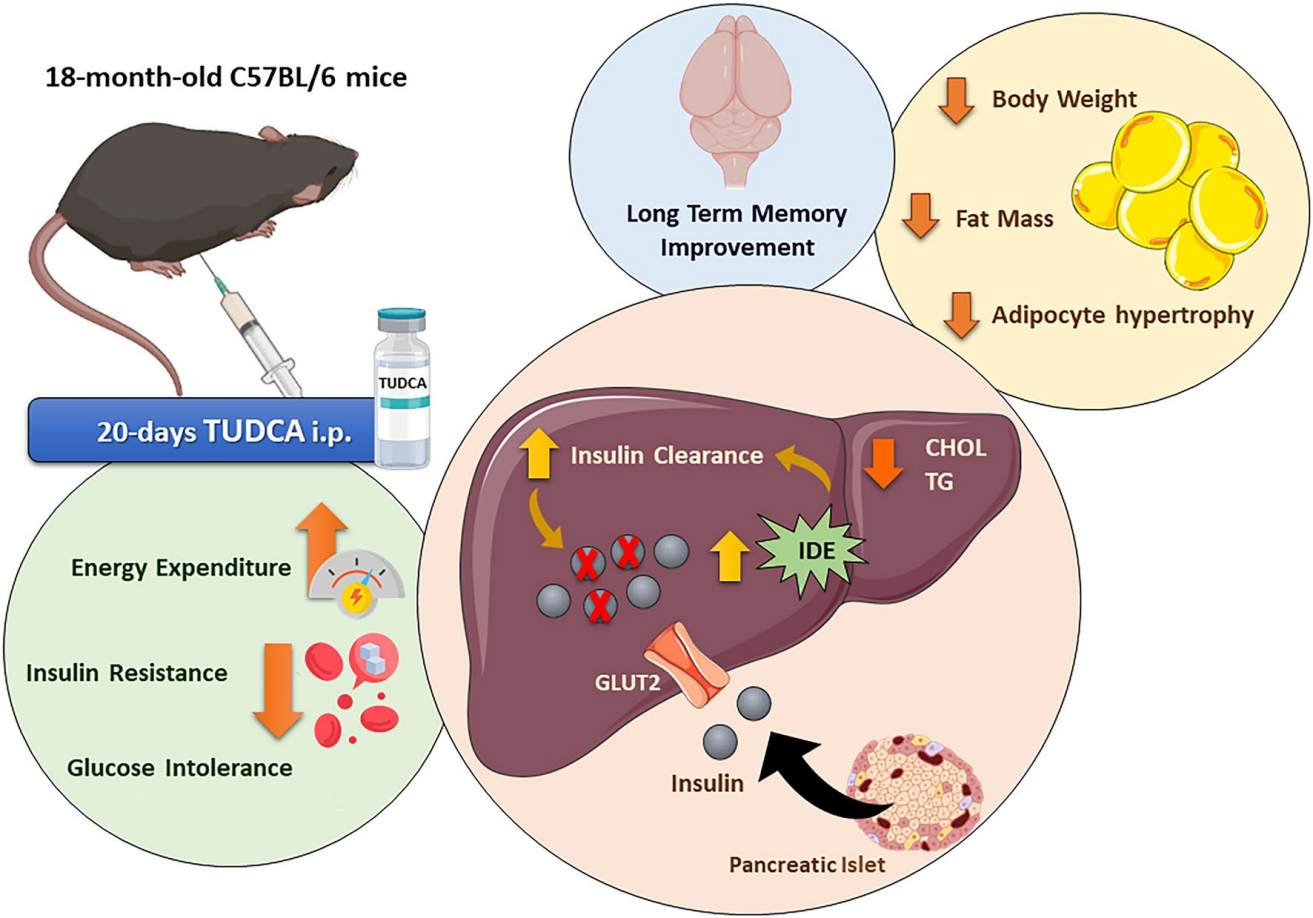
The bile acid TUDCA reduces age-related hyperinsulinemia and improves cognition in aged mice. TUDCA treatment for 20 days attenuated hyperinsulinemia and improved glucose homeostasis in 18-month-old mice, by enhancing liver IDE expression and insulin clearance. Furthermore, the improvement in glucose-insulin homeostasis in these mice was accompanied by a reduction in body weight, adiposity, and adipocyte hypertrophy, associated with reduced TG and CHOL levels in the liver. TUDCA-treated aged mice also displayed increased energy expenditure and ameliorated cognitive ability. In summary, these findings highlight TUDCA as an interesting target for the attenuation of age-related hyperinsulinemia and its deleterious effects on metabolism.

Lastly, to investigate whether the effects of TUDCA on energy homeostasis may also be dependent on modulations in the thermogenic capacity of BAT, we also went to evaluate this tissue. By using infrared thermography, we observed a lower BAT temperature in Old and Old + TUDCA mice, compared to Ctl mice (Fig. 7A,B), with no difference in tail temperature (Fig. 7C), evidencing that BAT temperature was not influenced by changes in body temperature. We also did not observe any significant difference in BAT weight between groups (Fig. 7D). Moreover, old and Old + TUDCA mice presented reduced expression levels of thermogenesis-related genes COX7A1, COX8B, DIO2, CIDEA, PPARGC1α, PRDM16, and UCP1 in BAT, compared to Ctl mice (Fig. 7E). These data suggest that BAT thermogenesis is not directly involved in ameliorated EE observed in Old mice treated with TUDCA (Fig. 8).

## Discussion

Over the last decades, the increase in life expectancy and progressive aging has been accompanied by a global epidemic of obesity and its related metabolic disorders, mainly T2D^22^. Dysfunction of the glucose-insulin homeostasis is widely recognized as a significant hallmark of the aging process that, in turn, results in systemic metabolic alterations^23^. These include insulin resistance, hyperinsulinemia, and accumulation of ectopic lipids, which are responsible for an elevated risk of obesity and T2D onset associated with aging^22^.

In this context, the bile acid TUDCA draws attention, since in addition to its beneficial effects on glucose and insulin metabolism^9,11,12,24^, it is a safe compound for humans when administered orally^7^. In this study, we evaluated the actions of TUDCA upon glucose-insulin homeostasis in 18-month-old male mice. We observed a reduction in body weight, adiposity, and hepatic lipid accumulation in Old + TUDCA mice, which also displayed an improvement in glucose tolerance, peripheral insulin sensitivity, and EE. These results were accompanied by lower insulinemia after glucose challenge, and higher hepatic gene and protein expression of IDE.

Several body modifications occur with aging, such as increased fat accumulation and muscle mass loss, both factors that impact the body weight^25,26^. These age-related changes in body composition are linked with increased insulin resistance and glucose intolerance^3,25,27,28^. Glucose intolerance is often associated with aging^23,29^, and elevated levels of both glucose and insulin have been observed in the elderly after oral glucose challenge testing^30^. Age-related hyperinsulinemia may be the consequence of an increase in insulin secretion and/or a decrease in its clearance^31^. It is known that hyperinsulinemia downregulates insulin receptors at the cellular membrane and disrupts post-receptor intracellular signaling in its target cells, inducing insulin resistance^32,33^. Insulin resistance is a key risk factor for T2D and is associated with a variety of intermediate phenotypes (hypertension, atherosclerosis, obesity) strongly affecting morbidity, disability, and mortality among the elderly^34^.

It was previously demonstrated that ablation of the insulin gene, in an age‐dependent and high‐fat diet‐induced hyperinsulinemia mice model, prevented diet‐induced obesity and its complications, such as insulin resistance and hyperinsulinemia^35^. Furthermore, genetic prevention of chronic hyperinsulinemia increased EE, reduced hepatic steatosis, and normalized the size of adipocytes in this model^35^. It has also been observed that reduced circulating insulin levels increased insulin sensitivity in aged mice, as well as extended their lifespan^36^. Corroborating these findings, blocking hyperinsulinemia with drugs, including diazoxide or octreotide, promotes weight loss in humans^35^, and in obese rats, via an increase in basal metabolic rate^37^.

We observed in TUDCA-treated Old mice similar insulin secretion compared to PBS-treated Old mice, judging by similar concentrations of c-peptide after glucose loading, and equal glucose-stimulated insulin secretion in isolated pancreatic islets. However, plasma insulin concentrations were found to be lower, 30 and 60 min after glucose load in the Old + TUDCA group, probably due to an increase in insulin clearance. Insulin clearance plays a crucial role in the regulation of plasma insulin^38^. Previous studies have demonstrated that different interventions that improve insulin signaling are associated with increased insulin clearance and IDE expression^11^. The liver is the main site for insulin degradation and can remove about 50–80% of insulin secreted during its first passage^39^, which occurs through the involvement of IDE^10,38^. It has already been observed that age-induced hyperinsulinemia is associated with a reduction in the hepatic expression e function of IDE^31^. Therefore, we evaluated the insulin clearance, as well as the protein content, gene expression, and the activity of the IDE in the liver of mice. Indeed, when the c-peptide/insulin ratio was calculated, insulin clearance was found to be higher in TUDCA-treated Old mice. We also observed an increase in IDE gene and protein expression in the liver of these mice, supporting our hypothesis that the attenuation of insulinemia by TUDCA is involved with increasing insulin clearance.

Although the actions of TUDCA upon insulin clearance in aged mice had not been investigated before, the effects of TUDCA in increasing liver IDE protein expression and insulin clearance have also been observed in diet-induced obese mice^11^, and this effect appears to be dependent on S1PR2 activation and its downstream proteins, including insulin receptor (IR), phosphatidylinositol 3-kinase (PI3K), and protein kinase B (AKT)^11,40^.

It is necessary to keep in mind that TUDCA can exert its actions through other receptors, such as Takeda G-protein receptor 5 (TGR5), α5β1 integrin, and farnesoid X receptor (FXR)^41^. The action of TUDCA through FXR seems to be controversial within the literature. Some studies point out that FXR has a low affinity for TUDCA (and its precursor, UDCA), due to hydrophilic nature of these bile acids^41^. However, in a presence of bile acid transporter, such as Na^+^-taurocholate co-transporting polypeptide (NTCP) in hepatocytes, TUDCA can enter cells and bind to the nuclear receptor FRX^42^.

We observed that TGR5, α5β1 integrin, and FXR were also expressed in the liver (Supplementary Fig. S2). However, neither aging nor TUDCA modulated the hepatic gene expression of the bile acid receptors mentioned above. In addition, the pattern of gene expression was similar between the groups, with α5β1 integrin and FXR receptors being more expressed than TGR5 and S1PR2 (Supplementary Fig. S2). The variety of TUDCA receptors expressed in the liver, as well as the simultaneous activation of different signaling pathways, makes difficult the identification of the specific mechanisms by which TUDCA potentiates IDE expression, and further studies are required to elucidate the molecular mechanisms involved.

It has been previously demonstrated that hyperinsulinemia can also increase fat accumulation and adipocyte size, leading to an overflow of free fatty acids that subsequently promotes fat accumulation in the liver and other tissues, resulting in systemic insulin resistance^35^. Thus, we believe that the higher body weight and fat depots observed in Old mice may be linked to age-related hyperinsulinemia and reduction in EE^3,25^, since no changes in food intake were detected. TUDCA treatment efficiently reduced body weight and adiposity in Old mice, as previously observed in diet-induced obese mice^11^, ob/ob mice^9^, and AD mice models^12,43^. Moreover, TUDCA also reduces the accumulation of lipids in the liver of obese mice, probably due to an improvement in insulin sensitivity^9^. Another important point is the ability of TUDCA and its precursor UDCA to reduce the absorption of cholesterol in the small intestine of mice^44,45^. Even though Old mice did not show changes in circulating cholesterol concentrations, in models where hypercholesterolemia is observed, such as in mice subjected to HFD, TUDCA was able to reduce the intestinal absorption of cholesterol and its circulating levels^45^.

One of the most important effects of insulin is to inhibit gluconeogenesis in the liver^46^. Hepatic insulin resistance can lead to elevated hepatic glucose production in aging^47^, so that glucose overload can be converted to TG, through de novo lipogenesis^48^. Besides that, impaired fatty acid oxidation in the liver can also contribute to the age-associated increase in hepatic lipids accumulation^48^. Therefore, we believe that the reduction in hepatic TG and CHOL observed in TUDCA-treated Old mice may be associated with the improvement in insulin sensitivity.

It is known that advancing age is also associated with cognitive decline, which predisposes individuals to neurological and psychiatric disorders, affecting the quality of life^49^. Among the disorders resulting from aging that affect cognition, AD is one of the major health problems of the elderly population^50^. Interestingly, we observed that Old mice treated with TUDCA presented better performance during a behavioral test, suggesting ameliorated memory formation ability. Furthermore, TUDCA has recently been shown to markedly reduce age-dependent amyloid accumulation in the mouse brain, the main marker of AD^43^. The capability of TUDCA to improve cognition and reduce memory loss has already been observed in different AD mice models^12,51,52^, that can be explained through two mechanisms: i, a direct action of TUDCA in the hippocampus, activating pathways that will reduce oxidative and ER stress, inflammation and apoptosis, increasing cell proliferation and survival^7^; and ii, an indirect effect of TUDCA on the central nervous system (CNS), as a consequence of its peripheral actions. We recently showed that the beneficial actions of TUDCA involved in delaying neurodegeneration in AD are not limited to the CNS, since the effects of TUDCA on peripheral tissues also reduce the main neuro markers of AD^12,13^. Thus, we believe that in our aged model, TUDCA actions associated with cognitive improvement may also involve central and peripheral signaling.

In summary, our data show an important role for TUDCA in reducing age-induced hyperinsulinemia and its complications, pointing this bile acid as an interesting therapeutic target for the treatment of glucose-insulin disturbance in the elderly. Our study is the first to show the therapeutic effects of TUDCA upon glucose homeostasis in aged mice, suggesting a novel therapeutic approach in the gerontology field.

## Supplementary Information

Supplementary Information.

## Data Availability

The datasets used and analyzed during the current study are available from the corresponding author on reasonable request.

## Abbreviations

AD: Alzheimer’s disease
ANOVA: One-way analysis of variance
AUC: Area under the curve
CIDEA: Cell death-inducing DNA fragmentation factor alpha-like effector A
CHOL: Cholesterol
CNS: Central nervous system
COX7A1: Cytochrome c oxidase subunit 7A1
COX8B: Cytochrome c oxidase subunit 8B
DIO2: Iodothyronine deiodinase 2
EE: Energy expenditure
ELISA: Enzyme-linked immunosorbent assay
ER: Endoplasmic reticulum
eWAT: Epididymal white adipose tissue
FXR: Farnesoid X receptor
GAPDH: Glyceraldehyde-3-phosphate dehydrogenase
GSIS: Glucose-stimulated insulin secretion
GTT: Glucose tolerance test
H&E: Hematoxylin and eosin
iBAT: Interscapular brown adipose tissue
IDE: Insulin-degrading enzyme
ITT: Insulin tolerance test
iWAT: Inguinal white adipose tissue
kITT: Constant rate for glucose disappearance
PBS: Phosphate buffer saline
PPARGC1α: Peroxisome proliferator-activated receptor gamma coactivator 1 alpha
PRDM16: PR/SET domain 16
prWAT: Perirenal white adipose tissue
RER: Respiratory exchange ratio
rpWAT: Retroperitoneal white adipose tissue
S1PR2: Sphingosine-1-phosphate receptor 2
TGR5: Takeda G-protein receptor 5
TUDCA: Tauroursodeoxycholic acid
TG: Triglycerides
T2D: Type 2 diabetes
UCP1: Uncoupling protein 1
UDCA: Ursodeoxycholic bile acid
