# Supplementary material for: The bile acid TUDCA reduces age-related hyperinsulinemia in mice
Source: Sci Rep. 2022 Dec 23;12:22273. doi: 10.1038/s41598-022-26915-3 (PMC9789133; doi:10.1038/s41598-022-26915-3)
Supplement: Supplementary file 1 — Supplementary Information. [file 41598_2022_26915_MOESM1_ESM.docx]

**The bile acid TUDCA reduces age-related hyperinsulinemia in mice**

Lucas Zangerolamo^1^, Marina Carvalho^1^, Leticia Barssotti^1^, Gabriela M. Soares^1^, Carine Marmentini^1^, Antonio C. Boschero^1^, Helena C. L. Barbosa^1*^

^1^ Obesity and Comorbidities Research Center, Department of Structural and Functional Biology, University of Campinas, UNICAMP, Campinas, Sao Paulo, Brazil.

Supplementary Table 1: Primer sequences for real-time qPCR assays

| **Gene** | **Forward (5’ – 3’)** | **Reverse (5’ – 3’)** |
| --- | --- | --- |
| IDE | CTGTGCCCCTTGTTTGATGC | GTTCCCCGTAGCCTTTTCCA |
| DIO2 | AATTATGCCTCGGAGAAGACCG | GGCAGTTGCCTAGTGAAAGGT |
| PPARGC1α | TATGGAGTGACATAGAGTGTGCT | CCACTTCAATCCACCCAGAAAG |
| CIDEA | TGCTCTTCTGTATCGCCCAGT | GCCGTGTTAAGGAATCTGCTG |
| PRDM16 | TGCTGACGGATACAGAGGTGT | CCACGCAGAACTTCTCGCTAC |
| COX7A1 | CAGCGTCATGGTCAGTCTGT | AGAAAACCGTGTGGCAGAGA |
| COX8B | TGTGGGGATCTCAGCCATAGT | AGTGGGCTAAGACCCATCCTG |
| UCP1 | CTGCCAGGACAGTACCCAAG | TCAGCTGTTCAAAGCACACA |
| S1PR2 | ATGGGCGGCTTATACTCAGAG | GCGCAGCACAAGATGATGAT |
| FXR | GCTTGATGTGCTACAAAAGCTG | CGTGGTGATGGTTGAATGTCC |
| α5β1 integrin | CTTCTCCGTGGAGTTTTACCG | GCTGTCAAATTGAATGGTGGTG |
| TGR5 | CCTGGAACTCTGTTATCGCTCA | GCACTCGTAGACACCTTTGGG |
| GAPDH | AGGTCGGTGTGAACGGATTTG | AGTAGACCATGTAGTTGAGGTCA |

IDE: insulin degrading enzyme; DIO2: iodothyronine deiodinase 2; PPARGC1α: peroxisome proliferator-activated receptor gamma coactivator 1 alpha; CIDEA: cell death-inducing DNA fragmentation factor alpha-like effector A; PRDM16: PR/SET domain 16; COX7A1: cytochrome c oxidase subunit 7A1, COX8B: cytochrome c oxidase subunit 8B, UCP1: uncoupling protein 1; S1PR2: sphingosine-1-phosphate receptor 2; FXR: farnesoid X receptor; TGR5: Takeda G-protein receptor 5; α5β1 integrin, and GAPDH: glyceraldehyde 3-phosphate dehydrogenase.

**Supplementary Figure 1**

**Membrane 01**


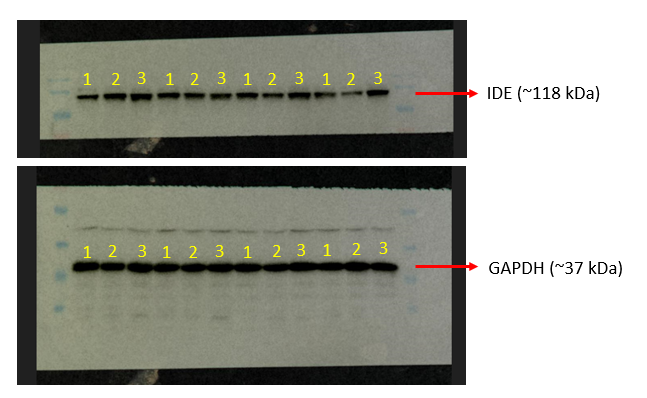


**Membrane 02**


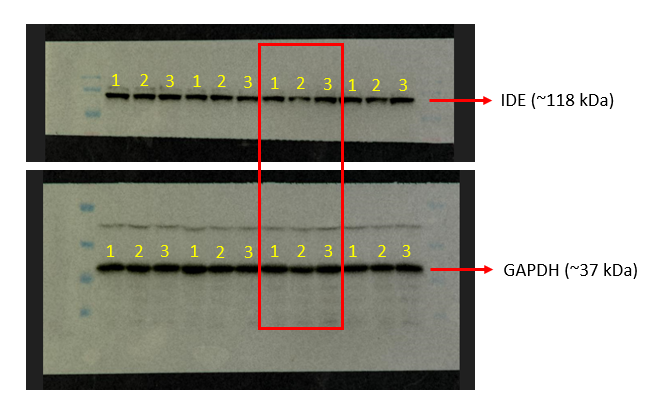


**Supplementary Figure 1: Original unprocessed blot images from Figure 4F.** Groups: Ctl (1), Old (2), Old+TUDCA (3), experimental number per group = 8. Full scan of the entire original gel incubated with IDE and GAPDH antibodies. Red Box indicate the representative image shown in Figure 4F. The samples were transferred to nitrocellulose membranes in this sequence: Ctl (1), Old (2) and Old+TUDCA (3).

**Supplementary Figure 2**


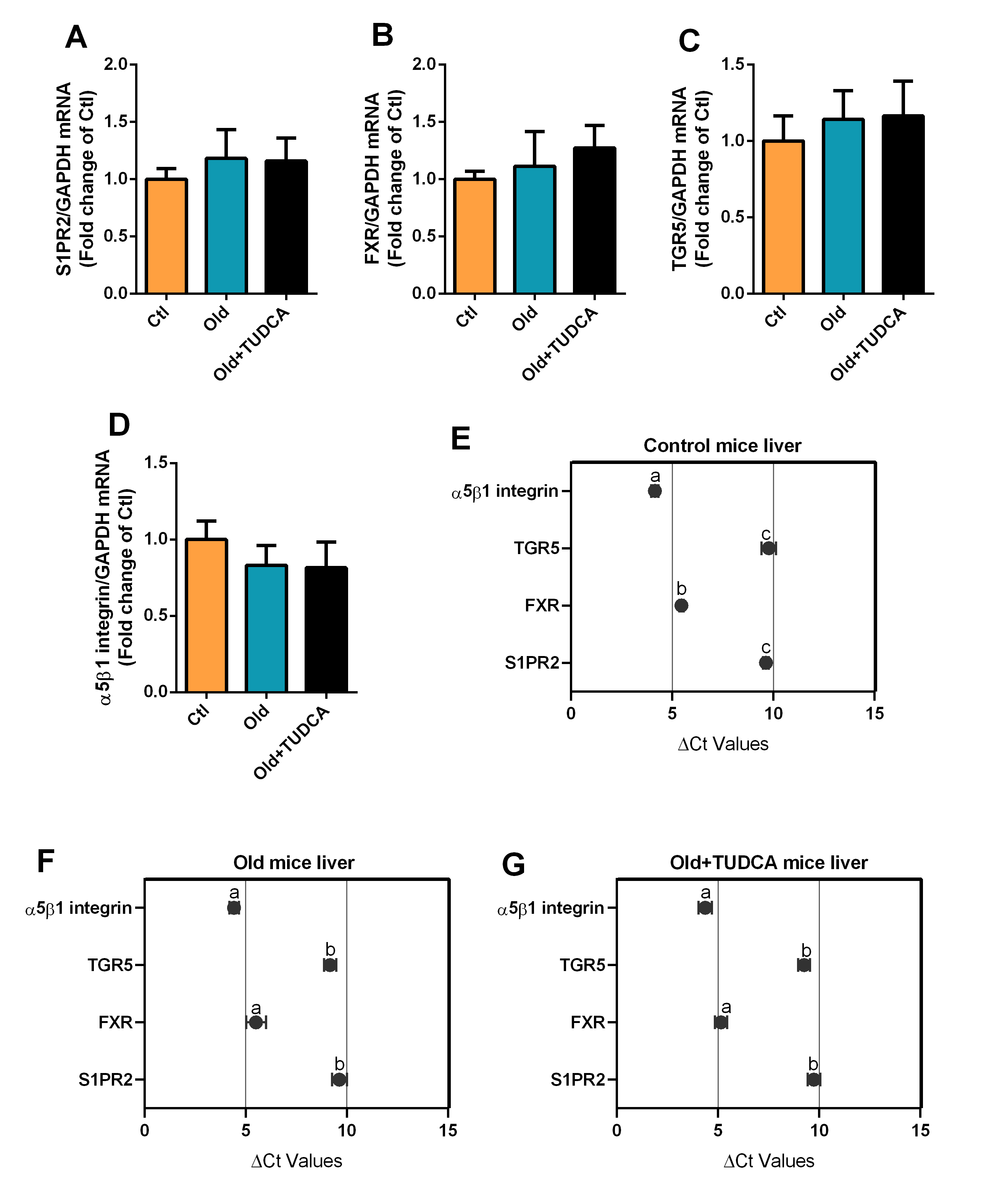


**Supplementary Figure 2: Effects of TUDCA treatment on hepatic bile acid receptor gene expression.** Gene expression of S1PR2 (A), FXR (B), TGR5 (C), and α5β1 integrin (D) in the liver, normalized by GAPDH. ΔCT values were averaged for each investigated gene over the Ctl (E), Old (F), and Old+TUDCA groups. The ΔCT values denote the normalized CT value, by subtracting the CT value of endogenous control GAPDH from the CT value of each investigated gene. The lower ΔCT value means the higher level of gene expression. Data are expressed as means ± SEM (n = 8-10). Statistical analysis was performed using the one-way ANOVA test, followed by Tukey post-hoc-test. Letters shared in common between groups indicate no significant difference. Different letters (a, b, and c) indicate statistical difference between groups (P ≤ 0.05).
